# Pattern of intentional drowning mortality: a total population retrospective cohort study in Australia, 2006–2014

**DOI:** 10.1186/s12889-019-6476-z

**Published:** 2019-02-19

**Authors:** Muthia Cenderadewi, Richard C. Franklin, Amy E. Peden, Sue Devine

**Affiliations:** 1 College of Public Health, Medical and Veterinary Sciences, James Cook University, Townsville, QLD 4811 Australia; 2 Royal Life Saving Society – Australia, Sydney, NSW 2007 Australia

**Keywords:** Intentional drowning, Suicide drowning, Drowning prevention, Health promotion, Australia, Epidemiology

## Abstract

**Background:**

While a downward trend in unintentional drowning deaths in Australia has been observed, little is known about intentional drowning mortality. Limited information on intentional drowning death impedes the planning, implementation, and evaluation of prevention strategies. This study aims to describe rates of intentional fatal drowning in Australia and compare these to other categories of drowning.

**Methods:**

Data were sourced from the Australian Bureau of Statistics (ABS) over a 9-year period (2006–2014). Rates and trends of intentional drowning were compared with unintentional, water-transport related and undetermined intent drowning. Rates of intentional drowning deaths across gender, age groups, states/territories, remoteness of residence and First Peoples of Australia were calculated. Relative risk (RR) (95% confidence interval [CI]) was calculated, and chi-square tests of independence were performed (*p* < 0.05).

**Results:**

The crude mortality rate for intentional drowning deaths in Australia over the study period was 0.23/100000, lower than unintentional drowning (0.89/100000). Males were 1.6 (CI: 1.4–2.0) times more likely than females to intentionally drown, however females made up a significantly larger proportion of intentional drowning deaths (38.2%) compared to unintentional deaths (22.4%) (χ^2^ = 47.3; df = 1; *p* < 0.05). A significant linear association between age group and intentional drowning was observed (χ^2^ = 131.3; *p* < 0.05), with individuals aged 75 years and over 32.6 times more likely to intentionally drown. Non-Indigenous peoples were 4.1 times more likely to intentionally drown in comparison to First Peoples of Australia. Residents of Inner Regional, Outer Regional, and Major Cities were 4.2 times (CI: 0.6–30.0), 4.1 times (CI: 0.6–29.9), and 4.0 times (CI: 0.6–28.6) more likely to intentionally drown, respectively, compared with residents of Very Remote areas.

**Conclusions:**

This study adds to the limited evidence currently available about intentional drowning rates and trends in Australia. Being male, of older age groups, non-Indigenous, residing in Inner and Outer Regional areas, and Major Cities were risk factors for intentional drowning deaths. Improving data collection systems and furthering understanding of the risk factors of intentional drowning, as well as the development, implementation, and evaluation of prevention programmes, are required to reduce the risk of intentional drowning death in Australia.

## Background

Drowning is a leading cause of mortality, morbidity, and disability across the world [1, 2]. An estimated 372,000 drowning deaths occur worldwide annually, although this is very likely to be under-estimated [1, 3, 4]. As a result of development and increased investment in the area of injury prevention and water safety regulation, a decreasing trend in the global incidence of unintentional drowning deaths has been observed [3, 5–8]. Despite this promising trend, less is known as to whether this downward trend in unintentional deaths is reciprocated by a decline in intentional drowning death rates [9, 10].

The published literature on the extent and trends of intentional drowning death is limited, even amongst high income countries [11–16]. However, even within the limited number of studies identified, a high proportion of intentional drowning deaths were observed [11–15]. A South Korean study revealed 74.2% of drowning cases admitted to emergency departments between 1998 and 2011 had drowned themselves intentionally, with the intentional drowning cases (median age: 35.0 years; range: 25.0–49.0 years) significantly older than those who had drowned unintentionally (median age: 26.5 years; range:19.0–35.5 years) (*p* < 0.001) [17]. A Swedish study over the period of 1992–2009 reported 31% of all drowning deaths in the country were identified as suicidal, with a significantly higher proportion in females (55%, *p* < 0.001) and the highest incidence in those aged 50–59 and 60–69 years [11].

Prevention is vital in reducing the mortality and disability caused by a drowning incident, including an intentional drowning [1, 18]. It has been proposed that examining all-intent drowning deaths is valuable, as measures used to prevent unintentional drowning may also be appropriate for intentional drowning; and due to the difficulty in determining intent of drowning fatalities [19]. Therefore, enhancing understanding of the magnitude of intentional drowning as a public health problem in Australia, by examining its rates and risk factors as compared to other categories of drowning, is critical to the planning, implementation and evaluation of intentional drowning prevention strategies.

To further extend the understanding of the magnitude of intentional drowning in Australia, this study aimed to examine the rates of fatal intentional drowning in Australia, as compared to other drowning classifications, over the period of 2006 to 2014, using data from the Australian Bureau of Statistics (ABS).

## Methods

### Data source

This study was undertaken as a total population retrospective cohort study. An analysis of quantitative, national data sourced from the ABS between 2006 and 2014 was performed. The study time period was used as at this was the most currently available data at the time of analysis. The inclusion criteria for intentional drowning was all death cases registered with the underlying cause of death of suicide and assault by drowning and submersion, based on the ICD-10 [20], which were coded as:

- ‘X71’ for intentional self-harm by drowning and submersion
- ‘X92’ for assault by drowning and submersion

The mortality rates over time were calculated for intentional drowning in Australia between 2006 and 2014, to be compared with three other categories of drowning death: unintentional, water-transport related and undetermined intent. Population data was sourced from the ABS to calculate drowning rates per 100,000 population [21]. The three comparison categories of drowning obtained in this study were coded by ICD-10 as:

1. Unintentional/accidental drowning deaths

- ‘W65’ for drowning and submersion while in bath-tub
- ‘W66’ for drowning and submersion following fall into bath-tub
- ‘W67’ for drowning and submersion while in swimming-pool
- ‘W68’ for drowning and submersion following fall into swimming-pool
- ‘W69’ for drowning and submersion while in natural water
- ‘W70’ for drowning and submersion following fall into natural water
- ‘W73’ for other specified drowning and submersion
- ‘W74’ for unspecified drowning and submersion

2. Water-transport related drowning deaths

- ‘V90’ for accident to watercraft causing drowning and submersion
- ‘V92’ for water-transport-related drowning and submersion without accident to watercraft

3. Undetermined intent drowning deaths

- ‘Y21’ for drowning and submersion, undetermined intent

Water-transport related drowning fatalities were examined separately from accidental drowning cases, just as classified by the ICD-10. The separation of the two categories of drowning is also due to the different prevention strategies needed.

Unit record data was provided by the ABS, however only a limited number of variables (*n* = 7) were provided. The following information was extracted for all four categories (intentional, unintentional, water-transport related and undetermined intent) of drowning deaths:

- Gender
- Age group
- State/territory of usual residence
- Postcode of usual residence
- Country of birth of deceased (classified into ‘Australia’ and ‘overseas’)
- Date of death
- First Peoples of Australia, in this study refers to “Aboriginal people”, “Torres Strait Islander people”, and “Aboriginal and Torres Strait Islander people”. Individuals who are not identified as these categories are classified as “non-Indigenous people” [22]

There were a further three variables that were derived from the seven variables provided by the ABS. Date of death was used to code season of death and day of week of death. Postcode of usual residence was used to code remoteness of usual residence. This was classified based on the Australian Statistical Geography Standard (ASGS) Remoteness Structure [23] into five categories: ‘Major Cities’, ‘Inner Regional’, ‘Outer Regional’, ‘Remote’, and ‘Very Remote’. Season of death, Day of week of death, and Country of birth were investigated for intentional and unintentional drowning deaths only.

### Analysis

Data extracted from the ABS was entered into a database in IBMSPSS Statistics V.24. Relative risk (RR) with a 95% confidence interval (CI) was calculated from the comparison of mortality rates across groups of variables of interest, to measure the association between exposure of interest (e.g. gender, age group, state /territory of usual residence, remoteness of usual residence and Indigenous identification) and the categories of (intentional, unintentional, water-transport related and undetermined intent) drowning deaths. Variables of interest were chosen due to their availability in the ABS data.

Chi-square tests of independence were undertaken to determine the significance of association between exposures, and the incidence of deaths by intentional and unintentional drowning. A chi-square test for trend was undertaken for measuring the linear trend between age group and intentional drowning death. Statistically significant differences were deemed at *p* < 0.05. Where chi-square analysis was undertaken multiple times within a variable, a modified Bonferonni post-hoc test was used to investigate the significant difference between comparison groups. A linear regression was used to explore trends over time. Categories of drowning with case counts of less than five were combined with similar categories with larger counts to avoid information that could potentially be identifying or disclose information not in the public domain, as per ethical requirements.

### Ethics

Ethical approval was obtained from the Human Research Ethics Committee of James Cook University (Ethics Approval Number H6136).

## Results

Of the 2730 drowning deaths in Australia between 2006 and 2014, 450 deaths (16.5%) were reported as intentional (Table 1**)**. The majority of drowning deaths in Australia over the study period were unintentional (64.7%; *n* = 1765) (Table 1). There were 6.3% (173 deaths) of drowning cases reported with undetermined intent (Table 1). A further 342 cases (12.5%) were water-transport related drowning deaths (Table 1).

**Table 1:** Number and crude mortality rate of drowning deaths by category, Australia by year and overall, 2006–2014

Year	Intentional drowning		Unintentional drowning		Water-transport related drowning		Undetermined intent drowning		All drowning deaths	
	Number of deaths	Mortality rate (per 100,000)^a^	Number of deaths	Mortality rate (per 100,000)^a^	Number of deaths	Mortality rate (per 100,000)^a^	Number of deaths	Mortality rate (per 100,000)^a^	Number of deaths	Mortality rate (per 100,000)^a^
2006	46	0.22	209	1.02	27	0.13	16	0.08	298	1.46
2007	47	0.23	191	0.92	23	0.11	25	0.12	286	1.37
2008	48	0.23	184	0.87	20	0.09	32	0.15	284	1.34
2009	54	0.25	201	0.93	37	0.17	21	0.10	313	1.44
2010	46	0.21	221	1.00	34	0.15	21	0.10	322	1.46
2011	48	0.21	167	0.75	90	0.40	17	0.08	322	1.44
2012	50	0.22	194	0.85	49	0.22	8	0.04	301	1.32
2013	58	0.25	212	0.92	44	0.19	11	0.05	325	1.40
2014	53	0.23	186	0.79	18	0.08	22	0.09	279	1.19
Total	450	0.23^b^	1765	0.89^b^	342	0.17^b^	173	0.09^b^	2730	1.38^b^

Data Source: ABS Cause of Death Unit Record File 2006–2014

^a^The population of Australia used as the denominators in calculating the rates were derived from ABS data [21]

^b^The crude mortality rate for the period of 2006–2014 (per 100,000)

Of intentional drowning deaths, 96.7% (*n* = 435) were self-harm and 3.3% (*n* = 15) were registered as assault (Table 2). Fifty percent (225 cases) of self-harm drowning incidents took place in other specified water bodies, with no further information on the exact water body of the location of death available in the ABS register (Table 2).

**Table 2:** Intentional drowning deaths in Australia by ICD 10 code between 2006 and 2014

ICD 10 codes	n	%	Explanation
Intentional self-harm by drowning			
X71	118	26.2	Intentional self-harm by drowning and submersion
X71.0	81	18.0	Intentional self-harm by drowning and submersion while in bathtub
X71.1-X71.3	5	1.1	Intentional self-harm by drowning and submersion while in swimming pool, after jumping into swimming pool, and in natural water
X71.8	225	50.0	Intentional self-harm by drowning and submersion in other specified water
X71.9	6	1.3	Intentional self-harm by drowning and submersion in unspecified water
Total	435	96.7	
Assault by drowning			
X92, X92.0. X92.8	15	3.3	Assault by drowning and submersion in bathtub and in other specified water
Total for all intentional drowning deaths	450	100.0	

Data Source: ABS Cause of Death Unit Record File 2006–2014

### The rates and trends of drowning deaths in Australia between 2006 and 2014

**Fig. 1:**
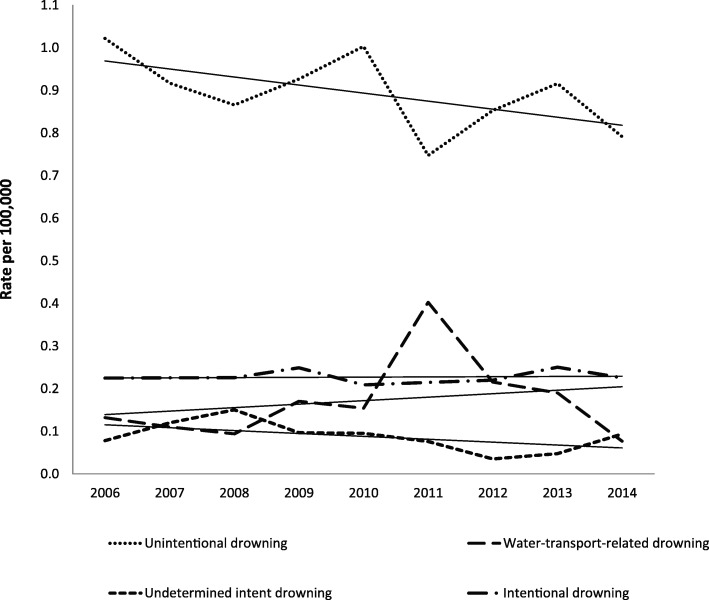
Trends in drowning deaths in Australia between 2006 and 2014

The crude mortality rate (CMR) for all drowning deaths in Australia over the period of 2006 to 2014 was 1.38/100000 (Table 1). The CMR for intentional drowning death over the study period was 0.23/100000, lower than for unintentional drowning of 0.89/100000 (Table 1). There was a downward trend in the unintentional drowning death rate (y = − 0.0188x + 0.9878), from 1.02 in 2006 to 0.79/100,000 in 2014. The intentional drowning death rate trend was stable (y = 0.0005x + 0.2247) over the observed period of 9 years (Fig. 1**)**. Similar trends were also observed for undetermined intent (y = − 0.0068x + 0.1221) and water-transport related drowning (y = 0.0082x + 0.1308), except for 2011 when a spike in the annual mortality rate of water-transport related drowning was observed (Fig. 1).

### Socio-demographic characteristics as risk factors for fatal intentional drowning

In Australia, males were 1.6 times (CI: 1.4–2.0) more likely than females to intentionally drown. However, this risk is comparatively lower than for other categories of drowning, with males 3.5 (CI: 3.1–3.9), 5.6 (CI: 4.2–7.6), and 2.6 (CI: 1.9–3.7) times more likely to drown as a result of unintentional drowning, water-transport related drowning, and undetermined intent drowning respectively when compared to females (Table 3). Females made up a significantly larger proportion of intentional drowning deaths (male: 61.8%, *n* = 278; female: 38.2%, *n* = 172) compared to unintentional drowning deaths (male: 77.6%, *n* = 1370; female: 22.4%, *n* = 395) (χ^2^ = 47.3; df = 1; *p* < 0.05) (Table 3).

**Table 3:** Number, rate and relative risk of socio-demographic characteristics of drowning deaths by category, Australia, 2006–2014

	Intentional drowning			Unintentional drowning			Intentional drowning versus unintentional drowning	Water-transport related			Undetermined intent		
	Total cases (*N* = 450)	Annual mortality rate per 100,000	Relative Risk (CI)	Total cases (*N* = 1765)	Annual mortality rate per 100,000	Relative Risk (CI)	χ^2^ (*p*-value)	Total cases (*N* = 342)	Annual mortality rate per 100,000	Relative Risk (CI)	Total cases (*N* = 173)	Annual mortality rate per 100,000	Relative Risk (CI)
Sex													
Male	278	0.28	1.6 (1.4–2.0)	1370	1.39	3.5 (3.1–3.9)	47.3 (*p* < 0.001)^a^	290	0.29	5.6 (4.2–7.6)	125	0.13	2.6 (1.9–3.7)
Female	172	0.17	1*	395	0.40	1*		52	0.05	1*	48	0.05	1*
Age Group													
0–4 years	6	0.02	1*	213	1.65	1*	53.1 (*p* < 0.001)^b^	9	0.07	1*	5	0.02	1*
5–14 years		0.01	0.5 (0.1–2.6)	88	0.35	0.2 (0.2–0.3)	17.1 (*p* < 0.001)^b^	15	0.06	0.9 (0.4–2.0)		0.01	0.8 (0.1–4.7)
15–24 years	22	0.08	3.5 (1.0–11.6)	203	0.75	0.5 (0.4–0.6)	17.2 (*p* < 0.001)^b^	49	0.18	2.6 (1.3–5.3)	19	0.07	4.5 (1.1–19.3)
25–34 years	43	0.15	6.5 (2.0–21.0)	265	0.93	0.6 (0.5–0.7)	8.9 (*p* = 0.002)^b^	55	0.19	2.8 (1.4–5.6)	19	0.07	4.3 (1.0–18.6)
35–44 years	52	0.18	7.9 (2.5–25.4)	205	0.73	0.4 (0.4–0.5)	0.001 (*p* = 1.000)	69	0.24	3.5 (1.8–7.0)	26	0.09	6.0 (1.4–25.1)
45–54 years	86	0.32	13.8 (4.4–43.5)	207	0.77	0.5 (0.4–0.6)	17.0 (*p* < 0.001)^b^	52	0.19	2.8 (1.4–5.6)	28	0.10	6.7 (1.6–28.2)
55–64 years	81	0.36	15.6 (4.9–49.4)	214	0.96	0.6 (0.5–0.7)	10.7 (*p* = 0.001)^b^	41	0.18	2.6 (1.3–5.4)	30	0.13	8.7 (2.1–36.3)
65–74 years	66	0.45	19.3 (6.1–61.3)	163	1.11	0.7 (0.6–0.8)	11.4 (*p* = 0.001)^b^	32	0.22	3.1 (1.5–6.5)	21	0.14	9.2 (2.2–39.3)
75 years and over	94	0.76	32.6 (10.3–102.8)	207	1.67	1.0 (0.8–1.2)	25.6 (*p* < 0.001)^b^	20	0.16	2.3 (1.1–5.1)	25	0.20	13.0 (3.1–54.8)
State/territory of usual residence													
New South Wales	131	0.20	1.7 (0.6–4.5)	599	0.93	1.4 (0.9–2.1)	3.8 (*p* = 0.056)	76	0.12	1.3 (0.4–4.1)	74	0.12	1.9 (0.5–7.7)
Victoria	98	0.20	1.6 (0.6–4.4)	265	0.54	0.8 (0.5–1.2)	12.1 (*p* = 0.001)^c^	27	0.05	0.6 (0.2–2.0)	43	0.09	1.4 (0.4–5.9)
Queensland	96	0.24	2.0 (0.7–5.4)	432	1.09	1.6 (1.1–2.5)	2.0 (*p* = 0.173)	76	0.19	2.1 (0.7–6.6)	20	0.05	0.8 (0.2–3.5)
South Australia	27	0.18	1.5 (0.5–4.3)	92	0.63	0.9 (0.6–1.5)	0.4 (*p* = 0.484)	18	0.12	1.3 (0.4–4.6)	13	0.09	1.5 (0.3–6.4)
Western Australia	66	0.32	2.6 (1.0–7.2)	224	1.08	1.6 (1.0–2.5)	1.2 (*p* = 0.273)	110	0.53	5.8 (1.8–18.2)	9	0.04	0.7 (0.2–3.3)
Tasmania	24	0.53	4.3 (1.5–12.4)	66	1.45	2.2 (1.3–3.5)	2.3 (*p* = 0.140)	25	0.55	6.0 (1.8–19.8)	14	0.26	4.3 (1.0–19.2)
Northern Territory	8	0.19	1.6 (0.4–6.4)	64	3.12	4.6 (2.9–7.5)	9.0 (*p* = 0.001)^c^	10	0.34	3.7 (1.0–14.3)		0.00	0.0 (UTBC)
Australian Capital Territory		0.18	1*	22	0.67	1*	0.4 (*p* = 0.632)		0.09	1*		0.06	1*
Remoteness of usual residence													
Major Cities	314	0.23	4.0 (0.6–28.6)	971	0.70	0.3 (0.2–0.4)	32.1 (*p* < 0.001)^e^	122	0.09	0.2 (0.1–0.3)	97	0.07	0.6 (0.2–2.5)
Inner Regional	86	0.24	4.2 (0.6–30.0)	338	0.93	0.4 (0.3–0.6)	0.0 (*p* = 1.000)	63	0.17	0.3 (0.2–0.7)	39	0.11	0.9 (0.2–3.9)
Outer Regional	45	0.23	4.1 (0.6–29.9)	229	1.27	0.6 (0.4–0.8)	4.4 (*p* = 0.036)	49	0.27	0.5 (0.3–1.1)	29	0.14	1.2 (0.3–5.2)
Remote		0.07	1.3 (0.1–14.0)	45	1.60	0.7 (0.5–1.1)	7.7 (*p* = 0.003)^e^	13	0.46	0.9 (0.4–2.1)		0.07	0.6 (0.1–4.5)
Very Remote		0.06	1*	40	2.26	1*	8.2 (*p* = 0.001)^e^	9	0.51	1*		0.11	1*
Indigenous identification													
First Peoples of Australia	NS	0.06	1*	97	1.96	1*	19.398 (*p* < 0.001) ^a^	11	0.22	1*	7	0.14	1*
Non-Indigenous	439	0.25	4.1 (1.3–12.7)	1629	0.91	0.5 (0.4–0.6)		289	0.16	0.7 (0.4–1.3)	163	0.09	0.7 (0.3–1.4)

Data Source: ABS Cause of Death Unit Record File 2006–2014

NS = Not Shown* Where RR was calculated, the group with the lowest rate (except for age groups, where the second lowest rate was used) for intentional drowning deaths was used as the reference point

^a^Statistically significant (*p* < 0.05)

^b^A modified Bonferroni test has been applied, and statistical significance is deemed at *p* < 0.0056

^c^A modified Bonferroni test has been applied, and statistical significance is deemed at *p* < 0.0063

^d^A modified Bonferroni test has been applied, and statistical significance is deemed at *p* < 0.0063

^e^A modified Bonferroni test has been applied, and statistical significance is deemed at *p* < 0.0100

A significant linear association between age group and the likelihood of intentional drowning was observed (χ^2^ = 131.3; *p* < 0.05) (Table 3). The mortality rates for intentional drowning deaths were higher with each increase in age group (Table 3). Using the youngest age group of 0–4 years as the reference, the highest risk of fatal intentional drowning was observed in the oldest age group of 75 years and over, with this group 32.6 times (CI: 10.3–102.8) more likely to intentionally drown (Table 3). The incidence of intentional drowning deaths among the youngest age group of 0–14 years was very low, and all are due to drowning by assault. The relative risks of drowning death with undetermined intent were similar to those observed in intentional drowning, with the risk peaking at the oldest age group of 75 years and older (Table 3). This trend is different from the risk of fatal unintentional drowning, with the highest annual mortality rate observed in the 0–4 years age group (1.65/100000) (Table 3). When comparing the proportion of deaths across age groups for intentional drowning and unintentional drowning, statistically significant differences were observed in all age categories (*p* = 0.006), except in the 35–44 years age group (Table 3).

The highest annual mortality rate for intentional drowning was registered in Tasmania (0.53/100000), and individuals with usual residence in Tasmania had 4.3 times (CI: 1.5–12.4) higher likelihood of dying from intentional drowning compared to the reference group, the Australian Capital Territory (ACT), which documented the lowest mortality rate of intentional drowning in Australia (Table 3). Individuals with usual residence in the Northern Territory (NT) were 4.6 times (CI: 2.9–7.5) more likely to die from unintentional drowning (Table 3). After modified Bonferroni tests were performed to compare the proportion of deaths across states/territories of usual residence in Australia, statistically significant differences were observed in two jurisdictions, Victoria and the NT (*p* = 0.006) (Table 3).

The highest rates of intentional drowning deaths by remoteness were identified in Inner Regional, Outer Regional, and Major Cities, with residents of these three remoteness categories having 4.2 times (CI: 0.6–30.0), 4.1 times (CI: 0.6–29.9), and 4.0 times (CI: 0.6–28.6) higher likelihood of intentionally drowning, in comparison to individuals in Very Remote areas (Table 3). This is the opposite for unintentional drowning deaths, with the highest mortality rate of 2.26/100000 identified in residents of Very Remote areas (Table 3).

Between 2006 and 2014, very few intentional drowning incidents were identified among First Peoples of Australia. Non-Indigenous peoples were 4.1 times (CI: 1.3–12.7) more likely to die from intentional drowning in comparison to First Peoples of Australia (Table 3). Higher risks for non-Indigenous peoples were also observed in water-transport related drowning deaths (RR = 1.4; CI: 1.8–2.5), a contrast to the unintentional and undetermined intent drowning deaths investigated, with non-Indigenous peoples 0.5 times (CI: 0.4–0.6) and 0.7 times (CI: 0.4–1.3) less likely to experience fatal unintentional and undetermined drowning death, respectively (Table 3).

For intentional drowning, the highest annual mortality rate was during summer (0.28/100000), although there was little seasonal variation observed (Table 4). Statistically significant differences were found for the proportions of intentional vs unintentional drowning during summer (χ^2^ = 10.6; df = 1; *p* = 0.013) and winter (χ^2^ = 18.8; df = 1; *p* = 0.013) (Table 4). For day of week of death, a significant difference was observed for the proportion of deaths due to intentional drowning (weekend days: 24.2%, *n* = 109; weekdays: 75.8%, *n* = 341) and unintentional drowning (weekend days: 35.0%, *n* = 618; weekdays: 65.0%, *n* = 1147) (χ^2^ = 18.9; df = 1; *p* < 0.05) (Table 4). Individuals born overseas were twice as likely to intentionally drown (RR = 2.0; CI: 1.7–2.4), but no significant difference was observed between fatal intentional drowning victims and unintentional drowning death cases in terms of country of birth (Table 4).

**Table 4:** Intentional and unintentional drowning by season, day of death, day of week and country of birth, Australia 2006–2014

	Intentional drowning			Unintentional drowning			Intentional drowning versus unintentional drowning
	Total cases (*N* = 450)	Annual mortality rate per 100,000	Relative Risk (CI)	Total cases (*N* = 1765)	Annual mortality rate per 100,000	Relative Risk (CI)	χ2 (*p*-value)
Season							
Summer	139	0.28	1.5 (1.1–1.9)	692	1.42	1.6 (1.5–1.9)	10.6 (*p* = 0.001)^a^
Autumn	95	0.19	1*	422	0.85	1*	1.6 (*p* = 0.236)
Winter	102	0.20	1.1 (0.8–1.4)	252	0.50	0.6 (0.5–0.7)	18.8 (*p* < 0.001)^a^
Spring	114	0.23	1.2 (0.9–1.6)	399	0.81	1.0 (0.8–1.1)	1.5 (*p* = 0.234)
Day of death							
Sunday	63	0.03	1.4 (0.9–2.0)	294	0.15	0.9 (0.8–1.1)	1.9 (*p* = 0.196)
Monday	75	0.04	1.6 (1.1–2.4)	211	0.11	0.7 (0.6–0.8)	7.1 (*p* = 0.009)
Tuesday	65	0.03	1.4 (1.0–2.1)	257	0.13	0.8 (0.7–0.9)	0.0 (*p* = 1.000)
Wednesday	76	0.04	1.7 (1.2–2.4)	229	0.12	0.7 (0.6–0.8)	4.6 (*p* = 0.038)
Thursday	65	0.03	1.4 (1.0–2.1)	236	0.12	0.7 (0.62–0.9)	0.4 (*p* = 0.539)
Friday	60	0.03	1.3 (0.9–1.9)	214	0.11	0.7 (0.6–0.8)	0.5 (*p* = 0.472)
Saturday	46	0.02	1*	324	0.16	1*	17.5 (*p* < 0.001)^b^
Day of week							
Weekend	109	0.19	1*	618	3.82	1*	18.9 (*p* < 0.001)^c^
Weekday	341	0.24	3.1 (2.5–3.9)	1147	1.14	1.9 (1.7–2.1)	
Country of birth							
Australia	261	0.18	1*	1070	0.18	1*	1.1 (*p* = 0.332)
Overseas	189	0.36	2.0 (1.7–2.4)	695	0.36	1.8 (1.6–2.0)	

Data Source: ABS Cause of Death Unit Record File 2006–2014

*Where RR was calculated, the group with the lowest rate was used as the reference point

^a^A modified Bonferroni test has been applied, and statistical significance is deemed at *p* < 0.013

^b^A modified Bonferroni test has been applied, and statistical significance is deemed at *p* < 0.007

^c^Statistical significance is deemed at *p* < 0.05

When the subsequent co-morbidities and causes detailed in the death certificate, including diseases, injuries and poisoning, were examined, a large frequency of underlying mental conditions were identified (32.0%; *n* = 144) among intentional drowning deaths. Of all mental disorders identified, depressive disorders were the most common (15.3%; *n* = 69), followed by mental and behavioural disorders due to the use of alcohol (5.3%; *n* = 24) and other anxiety disorders (11%; *n* = 2.4) (Table 5). Additional common co-conditions were poisoning (24.9%; *n* = 112), particularly by antiepileptic, sedative-hypnotic, and anti-parkinsonism drugs (8.9%; *n* = 40), psychotropic drugs (5.3%; *n* = 24) and due to toxic effect of alcohol (3.6%; *n* = 16), and intentional self-harm (13.1%; *n* = 59), which was mostly undertaken by self-poisoning as well (Table 5). A smaller proportion of cases (2.7%; *n* = 12) also registered symptoms and signs involving emotional state (coded by the ICD-10 as R45.8) (Table 5), however the documentation was not detailed enough as to infer the history of suicidal ideations (coded by the ICD-10 as R45.81).

**Table 5:** Common co-morbidities, conditions, and findings of the intentional drowning death victims, Australia, 2006–2014

Category		n	%
Mental disorders	Mental and behavioural disorders due to use of alcohol	24	5.3
	Mental and behavioural disorders due to use of other substances (tobacco, psychoactive substances, opioids, stimulants)	10	2.2
	Schizophrenia	5	1.1
	Bipolar affective disorder	10	2.2
	Depressive episode	69	15.3
	Other anxiety disorders	11	2.4
	Others (Schizoaffective disorders, unspecified nonorganic psychosis, recurrent depressive disorder, eating disorder, specific personality disorders, pervasive developmental disorders, hyperkinetic disorders, unspecified dementia, unspecified organic or symptomatic mental disorder, mental disorder not otherwise specified)	15	2.4
	Total	144	32.0
Emotional state	Other symptoms and signs involving emotional state	12	2.7
	Intentional self-poisoning by and exposure to antiepileptic, sedative-hypnotic, anti-parkinsonism, and psychotropic drugs, not elsewhere classified	20	4.4
	Intentional self-poisoning by and exposure to diuretics and other and unspecified drugs, medicaments, and biological substances	22	4.9
	Intentional self-poisoning by and exposure to others (non-opioid analgesics, antipyretics and anti-rheumatics, narcotics and psycho-dysleptics/hallucinogens not elsewhere classified, alcohol, gases and vapours)	10	2.2
	Intentional self-harm by sharp objects and jumping from a high place	7	1.6
	Total	59	13.1
Poisoning	Poisoning by non-opioid analgesics, antipyretics and anti-rheumatics	6	1.3
	Poisoning by narcotics and psycho-dysleptics/hallucinogens	9	2.0
	Poisoning by antiepileptic, sedative-hypnotic, and anti-parkinsonism drugs	40	8.9
	Poisoning by psychotropic drugs, not elsewhere classified	24	5.3
	Poisoning (Poisoning by diuretics and other and unspecified drugs, medicaments, and biological substances)	9	2.0
	Toxic effect of alcohol	16	
	Total	112	24.9
Finding of drugs and other substances not normally found in blood	Finding of alcohol in blood	18	4.0
	Finding of psychotropic drug in blood	22	4.9
	Finding of other substances (opiate drug, hallucinogen, steroid agent, and other drugs of addictive potential in blood)	10	2.2
	Total	50	11.1
Diseases of the nervous system	Alzheimer’s disease, Huntington’s disease, Parkinson’s disease, Epilepsy, Primary disorders of muscles, sleep disorders, other disorders of brain)	9	2.0

Source: ABS Cause of Death Unit Record File 2006–2014

## Discussion

Intentional drowning deaths in Australia represent 16.5% of all drowning deaths between 2006 and 2014. Intentional drowning is a little studied issue worldwide, including in Australia, and, while it represents a smaller proportion of drowning deaths in comparison to unintentional drowning, there has been no change in the Australian intentional rate of fatal drowning over the study period, therefore warranting focused attention.

### The rates and trends of intentional drowning in Australia: The comparison to unintentional drowning

The Crude Mortality Rate (CMR) for intentional drowning was 0.23/100000, lower than the CMR of unintentional drowning over the same period (0.89/100000) (Table 1). This mortality rate is also higher than rates described in the published literature on fatal intentional drowning in Australia, which reported annual rates of 0.06/100000 to 0.21/100000 between the period of 2000 to 2012 (Table 6**)** [24–27].

**Table 6:**
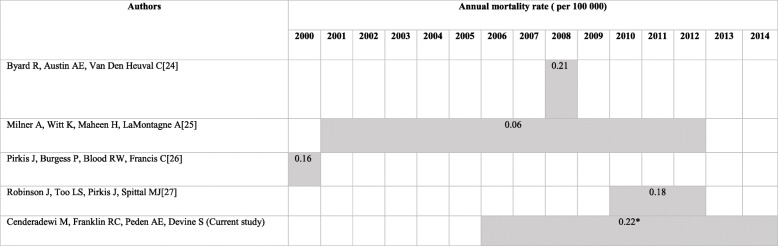
Annual nation-wide mortality rates of intentional drowning in Australia between 2000 and 2014

^*^Please note the rate in the current study has been amended slightly (from 0.23 to 0.22) to remove the 3.3% of intentional drowning deaths as a result of assault, to allow for comparison with the previously published studies which only examine suicidal drowning

The stable rate of intentional drowning in Australia (Fig. 1) does not correspond with the decreasing trend in suicide drowning in Australia over the period 1907–1998 reported by Donaldson et al. [28], from 3.10/100000 in 1907 to 1.00/100000 in 1998 among males, and from 1.25/100000 to 0.30/100000 in females, however the time period studied in this paper may not be long enough to see a downward trend. There are differences in rates of intentional drowning in Australia between this study and other published data presented in Table 6. The use of different data sources and study time periods, changes in data quality (i.e. closed vs open coronial cases) and population change may all impact rates presented. Further research is required to look at trends in intentional drowning over time.

The finding of a consistent trend of fatal intentional drowning in Australia is also dissimilar to other high income countries. A study in Sweden reported an average annual decrease of 2.00% between 1992 and 2009 (*p* < 0.001) [11] and a Norwegian study revealed an annual percentage change of female suicide drowning of an average − 3.70 between 1985 and 2012 (*p* < 0.05) [29]. Meanwhile, the stable rate over time in intentional drowning is comparable to the relatively stable rate over time of suicide deaths by all methods in Australia identified in a national-scale study over the period of 1986 and 2005 [30]. The complexity of changing classifications of undetermined cases over time may also impact trends in intentional and unintentional drowning deaths [8, 31].

This stable rate underlines the potential gap between the traditional focus of drowning prevention in Australia aimed at preventing the incidence of unintentional drowning deaths and a lack of focus on prevention of intentional drowning. The approach of unintentional drowning prevention has put more emphasis on water safety regulations, particularly physical access restrictions to water bodies and risk management of the hazards and risks associated with aquatic locations and activities [32–37]. Meanwhile, reducing the risk of intentional drowning events may require focusing on designing a comprehensive psychiatric assessment and management plan, by promoting identification, treatment and follow-up of individuals with psychiatric conditions to prevent a suicidal drowning attempt [11, 12, 32–36]. Education and training are also essential, particularly in promoting the impact of alcohol and substance misuse and training on immediate rescue and CPR by bystanders [11, 12]. Furthermore, regulating physical restriction to water bodies, such as bridge railings of appropriate height, has also been suggested as a part of intentional drowning prevention strategies [11, 12]. In addition, prevention programmes for intentional drowning need to specifically target older age groups [11, 12]. Such strategies require multi-disciplinary collaboration, involving key stakeholders across several government sectors such as public health, mental health, financial, social and welfare, and education services, to ensure drowning is included in the national suicide prevention strategy and its inclusion is supported by evidence-informed policy and practice at a national, regional and local level [36, 38].

### Intentional drowning risk factors in Australia: Informing the appropriate preventive measure for intentional drowning

The published literature on fatal intentional drowning in Australia identified being female [28], of older age [39], and substance use [40], as predictors for intentional drowning deaths, while underlining the lack of investigation into other potential risk factors for fatal intentional drowning in Australia. The examination of the 9-year ABS data source in the current study reveals males in older age groups, non-Indigenous people and residents of Inner Regional, Outer Regional, and Major Cities were more likely to intentionally drown, adding further information for the development of preventive interventions in Australia.

### Intentional drowning deaths in Australia by gender

In Australia, males were 1.6 time more likely to become fatal intentional drowning victims compared to females (Table 3). Dissimilar to this finding, a study in Croatia reported the higher proportion of suicide drowning death among females (17.49%), in comparison with suicide drowning found among males (4.66%) [41]. However, the higher risk of males intentionally drowning is consistent with several previous studies reporting higher rates of death by suicide observed amongst males in Australia [42–46]. A study by Burns [44] on suicide rates in Australia from 2004 to 2013 reported the highest suicide rate was observed amongst males aged 85 years and over with 33.44/100000, in comparison to the rate of 4.69/100000 among females from the same age group.

Studies on predictors of male suicide events among construction workers in Australia and on all work-related suicide in Victoria revealed inability to obtain steady employment, physical and social environment at work-place, financial and legal problems, family issues, a history of mental disorders, and substance abuse as contributing to the deaths [47, 48]. Therefore, interventions across an upstream, midstream, and downstream continuum are essential in reducing the risk of intentional drowning among males in Australia. Addressing inequities in various social determinants of health related to the risk factors for intentional self-harm in males, including employment, a healthy and supportive working environment, occupational risk management, social support network, rural and urban development, and the provision of mental health and education services, will be required [1, 49–52].

### Fatal intentional drowning amongst elderly Australians

A significant linear association between age group and the likelihood of intentional drowning was observed in comparison to unintentional drowning victims (χ2 trend = 131.3; *p* < 0.05) (Table 3). Individuals aged 75 years and over were 32.6 times more likely to die from intentional drowning (Table 3). This is similar to the finding of a study by Koo et al. [39] which reported the highest incidence of suicidal drowning in Australia in those aged 65–74 and 75–84 years, with the risk of intentional drowning significantly increasing with age (χ^2^ trend = 56.0, df = 1, *p* = 0.02), with individuals aged 65 to 74 years 0.7 (95% CI: 0.36–1.17) times less likely to perform suicide by drowning than individuals aged 75 to 84 years [39].

The higher risk of drowning amongst older age groups may be partially justified by aging-related cognitive deficits in the elderly, which potentially modify their preference for ‘less-violent’ suicide methods, as contended by a study by Purandare et al. [53], which reported self-poisoning and drowning as two of the most commonly used intentional death methods among patients with dementia in England and Wales. The high incidence of suicide events among older individuals may also be partially attributed to dementia [53–58].

### Urbanity as risk factor for intentional drowning

The highest rate of intentional drowning deaths were identified in Inner Regional, Outer Regional, and Major Cities, with residents of these three categories having 4.18 times, 4.12 times, and 4.01 times higher likelihood to die from intentional drowning, a contrast to unintentional drowning deaths, with the highest mortality rate identified in residents of Very Remote areas in Australia (Table 3). In urban areas in Queensland, a previous study reported the positive association between suicide rates and unemployment, median individual income, and households in public housing [59]. Therefore, in addition to investigating urbanity as a predictor for intentional drowning death, further research is required on the association between other socio-demographic characteristics, particularly socioeconomic status and unemployment with the incidence of intentional drowning death.

### Barriers in investigating intentional drowning deaths in Australia: Improving national data collection systems

This study has met its aims, including providing analysis on the rates, trends, and risk factors of intentional drowning in Australia between 2006 and 2014. Several strengths contributed to the robustness of this study, including the inclusion of all categories of drowning, and the use of a standardised, national-scale data source, to ensure all drowning deaths recorded by the ABS were included. However, several limitations were identified in this study, potentially related to barriers of intentional injury surveillance that have been identified in previous studies [11, 60–64].

### The challenging nature of investigating intentional death cases

First of all, determining the manner of deaths by suicide and assault can be challenging [11, 60–64]. As such this paper explores all intentional drowning deaths noting that many drowning-related intentional death studies only examine suicide [24–27]. Several studies [65–69] reported the complicated medico-legal investigation required to differentiate suspected suicide death and death by undetermined intent, which includes analysis of age, gender, socioeconomic status, history of mental disorders, history of suicide attempts and suicide ideations, circumstances at the scene of death, and toxicological and histopathological findings [70]. Therefore caution should be used when examining the cases coded as undetermined intent [71]. Challenges also exist around identifying co-morbidities in unnatural deaths, with studies indicating severe underreporting [71]. As such, the findings of this study with respect to medical conditions, should be interpreted with caution.

In addition, the legal framework of medico-legal investigation of death available in the country or state will implicate the investigation of unnatural death cases, as contended by a study by Walter et al. [72] on the characteristics of discretionary inquests for unnatural deaths in Australia between 2000 and 2007. Of all external deaths documented in Australia over the study period, only 6.1% proceeded to judicial inquest, and more than half of this proportion was discretionary [72]. Although the study reported that deaths by drowning, choking or suffocation were 5.12 times (95% CI: 3.56–7.39) more likely to move forward to discretionary inquest, it also highlighted the different statutory rules on regulating further investigation on certain types of deaths across jurisdictions in Australia [72]. Inconsistent coronial processes for determining intent of unnatural death cases, as a result of inadequate information and apprehension for labelling a case as suicide death due to social stigma, has also been suggested as one of the systemic reasons behind the under-counting of suicide deaths in Australia [31]. Therefore, further research on the development of a nationally consistent legal framework and medico-legal investigation of unnatural death cases is needed to improve data collection systems for intentional drowning deaths in Australia.

### The availability of a functioning national registration system with appropriate level of detail

The availability of a functioning national demographic, death, and medico-legal registration system is essential to ensuring quality data on intentional drowning in every country [11, 60–64, 73]. The nation-wide coverage of Australia’s national demographic, death, and medico-legal registration system, such as through the ABS and the National Coronial Information System (NCIS), contributes to the quality of intentional drowning data in Australian populations [74]. However, the potential of under-representation of fatal drowning in Australia related to the use of the ICD-10, the most widely-used coding framework to document deaths worldwide, needs to be explored in future research [4].

The ICD-10 coding combinations separate accidental drowning from intentional drowning by suicide, assault by drowning, drowning cases related to cataclysmic events, water transport incidents, and drowning events with undetermined intent [4, 75]. This current study identifies 6.34% (173 deaths) of drowning cases in Australia between 2006 and 2014 as drowning deaths with undetermined intent, outlining the possibility of the under-representation of intentional drowning statistics in Australia, as has been found for unintentional fatal drowning [4]. This can lead to misleading allocation of financial investment, public resources and policies for drowning prevention, both intentional and unintentional.

In addition, it is essential for a standardised, national coding system to be designed with an appropriate level of detail to properly document and report causes and circumstances surrounding death events, in order to ensure the comprehensiveness of intentional drowning data [11, 60–64, 73]. It is noted in this current study that more detailed information on co-morbidities, location, circumstances and toxicological and histopathological findings surrounding the event of death itself, needs to be documented in the standardised system of ABS registrations, although the NCIS provides some of this level of detail. Bugeja et al. [74] argued that the NCIS was a reliable source of medico-legal data with comprehensive coverage, although there were concerns about open cases and missing information which brought the potential for selection and reporting biases.

The availability of a functioning national demographic, death, and medico-legal registration system with an appropriate level of detail will enable the investigation of the association between intentional drowning events and underlying medical conditions, psychopathology, such as the history of mental disorders and the history of previous suicide attempts, substance use and dependence, and the location and activities related to intentional drowning in Australia. A better understanding of these risk factors will contribute to the development of public policy and the enhancement of community action in creating a supportive physical and social environment for intentional drowning prevention, along with mental health promotion, in Australia.

### Recommendations for future research

**Fig. 2:**
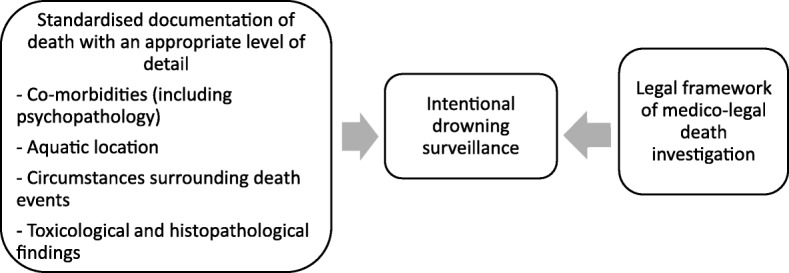
Future research needed for refining intentional drowning data collection systems

Coordinated, focused research on intentional drowning prevention is needed in Australia, consisting of the following: Improving the understanding of the magnitude of intentional drowning deaths by refining data collection systems (Fig. 2); providing a better comprehension of risk factors of intentional drowning death; and developing and evaluating prevention strategies..

**Fig. 3:**
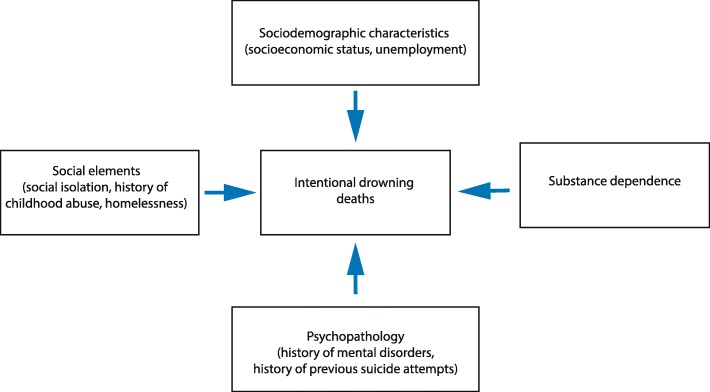
Risk factors of intentional drowning deaths needed to be investigated in future research for Australian context

Further investigation on several other risk factors, which have been suggested by previous studies as important predictors for suicide attempts, are needed to inform the planning, implementation, and evaluation of preventive interventions focused on reducing the risk of intentional drowning deaths in Australia. These may include: 1) socio-demographic characteristics, including socioeconomic status [40] and unemployment [40, 76]; 2) substance dependence [77]; 3) social elements [77], including history of childhood abuse [78], social isolation [79], and homelessness [80]; and 4) psycho-pathology, including depressive disorders [76, 77], psychotic disorders [77], anxiety disorders [77], and previous intentional self-harm attempts [12, 81–84]. A more comprehensive understanding on the risk factors of fatal intentional drowning, as illustrated in Fig. 3, will contribute to the development of supportive public policies and community participation for mental health and drowning prevention promotion in Australia.

Intentional drowning prevention and mental health promotion strategies need to be designed within the social-ecological framework, to ensure sustainability. A recent article [85] on the prevention of suicide and related self-injuries proposes a comprehensive public health approach to suicide prevention, aiming to introduce prevention interventions at individual, relationship, community and societal levels, which will be beneficial to be studied in the context of intentional drowning prevention.

Evaluating preventive interventions developed to reduce the risk of intentional drowning deaths in Australia is necessary, best conducted by undertaking sustained analysis of the rates and trend of intentional drowning in Australia.

## Conclusions

This study adds to the limited evidence currently available about intentional drowning, particularly its rates and trends in Australia. Being male, of older age groups, non-Indigenous, residing in Inner Regional, Outer Regional, and Major Cities were recognised as risk factors for intentional fatal drowning. Further research on improving data collection systems and understanding the risk factors for intentional drowning, as well as the development, implementation, and evaluation of prevention programmes, are required to reduce the incidence of intentional drowning deaths in Australia. A multi-disciplinary collaboration between public health and mental health institutions, education services, drowning prevention organisations, and the public, is required to achieve a coordinated effort to prevent intentional death by drowning.

## Abbreviations

ABS: Australian Bureau of Statistics
ACT: Australia Capital Territory
ASGS: Australian Statistical Geography Standard
CI: Confidence Interval
CMR: Crude mortality rate
CPR: Cardiopulmonary resuscitation
ICD-10: International Statistical Classification of Diseases and Related Health Problems 10th Revision
NCIS: National Coronial Information System
NT: Northern Territory
RR: Relative risk
UK: United Kingdom
